# Identification and Treatment of Catatonia Presenting as Agitation and Self Injury in an Adolescent With Rett Syndrome

**DOI:** 10.1155/crps/9715900

**Published:** 2025-09-29

**Authors:** Asim Al-Omari, Sarah Mohiuddin

**Affiliations:** Department of Psychiatry, University of Michigan, Ann Arbor, Michigan, USA

## Abstract

Catatonia is a complex psychomotor syndrome associated with several psychiatric disorders, including schizophrenia and autism. It is also associated with neurologic conditions such as encephalitis and epilepsy. Catatonia has also been described in genetic syndromes such as Down Syndrome. Catatonia presents with two main subtypes. Retarded catatonia is characterized by stupor, immobility, mutism, rigidity, and withdrawal, as well as negativism, posturing, and echolalia/echopraxia. Excited catatonia is primarily characterized by psychomotor agitation and occasionally self-injurious behaviors. Though the pathophysiology of catatonia remains poorly understood, treatment with benzodiazepines is effective in many cases, with electroconvulsive therapy indicated in cases of poor response. Rett syndrome is an X-linked neurodevelopmental disease associated with mutations in methyl-CpG-binding protein 2 and is characterized by regression of spoken language and purposeful hand skills, gait abnormalities, and stereotyped hand movements. Herein we describe a case of catatonia associated with Rett syndrome in a 17-year-old female. Her presentation was notable for hyperactivity, impulsive behaviors, agitation, self-injurious behaviors, and aggression. The patient had limited response to multiple medication trials. Initially she had a positive response to treatment with lorazepam, with later waning efficacy despite dose escalation. The patient was admitted to the inpatient psychiatry and completed an index course of 13 ECT treatments followed by maintenance ECT, completing 30 treatments in total. Treatment resulted in significant improvements in self-injurious behaviors, agitation, and overall engagement. In conclusion, catatonia should be considered in individuals with Rett syndrome who present with agitation and self-injury to aid in overall symptom improvement and outcome.

## 1. Introduction

Catatonia is a complex psychomotor syndrome associated with several psychiatric disorders including schizophrenia and autism. It is also associated with neurologic conditions such as encephalitis and epilepsy. Catatonia has also been described in genetic syndromes such as Down Syndrome. Rett syndrome is an X-linked neurodevelopmental disease associated with mutations in methyl-CpG-binding protein 2 and is characterized by regression of spoken language and purposeful hand skills, gait abnormalities, and stereotyped hand movements [1]. Herein we describe a case of catatonia associated with Rett syndrome in a 17-year-old female with an initial response to lorazepam who then had significant and last improvement with an index course of 13 ECT treatments followed by an extended course of ECT 3 x/weekly, completing 30 treatments in total.

### 1.1. Case Report

The patient is a 17-year-old female with a history of Rett Syndrome, autism spectrum disorder, intellectual disability, and severe receptive-expressive language disorder. Her baseline presentation was notable for hyperactivity, intermittent screaming, and impulsive behaviors, but was able to attend school and engage in tasks. Management included ongoing intensive behavioral intervention (applied behavioral analysis).

At approximately age 14, around the onset of puberty, the patient had significant and progressive worsening of symptoms, including constant screaming, multiple daily episodes of aggression, anger, and irritability, as well as the onset of motor and vocal stereotypies, self-injurious behavior, and loss of engagement and purposeful activities. Patient was trialed on multiple medications with limited response including antipsychotics (risperidone 0.5 mg BID over 2 years which initially helpful but later ineffective and led to weight gain and higher dosage lead to crawling, incontinence, and increased screaming, aripiprazole 2 mg dailyover 1 year with limited effectiveness, ziprasidone 20 mg TID with initial improvement that did not last), stimulants (Adderall, Vyvanse, Ritalin, and concerta all of which led to worsening agitation and aggression), antidepressants (sertraline for over 1 year 100 mg daily, worsened mood in the past, citalopram for 1 year 10 mg daily, ineffective, suspected to cause mood lability and decreased sleep, NAC: 2400 mg over 2 years with increasing severity of outbursts at the higher dose, trazodone100 mg nightly for several years), Mood stabilizers (carbamazepine used early childhood with excess sedation and valproic acid up to 375 mg BID with abrupt worsening of behavior- slapping and hitting others), Alpha agonists (guanfacine/intuniv1mg daily for several years, clonidine/Kapvay 0.2 mg nightly for several years).

Catatonia was suspected given the repetitive nature of screaming, increase in motor stereotypies from baseline, and loss of overall engagement. The patient was initiated on lorazepam and titrated up to 5 mg every 5 h (25 mg daily), which initially aided in reducing the severity, intensity, and frequency of these behaviors. It was noted that as treatment continued, the efficacy of lorazepam waned despite increasing doses.

The patient was admitted to the inpatient psychiatry unit and was noted to have elevated scores (15) on the Bush-Francis Catatonia Rating Scale on admission. CXR, MRI brain, and EEG were completed as a part of the initial workup for ECT. During the course of the hospitalization, she completed a course of 15 bilateral ECT treatments over 1 month. The medication regimen on admission included Lorazepam 5 mg five times daily.

Trazodone 100 mg at bedtime, and trofinetide 10,000 mg twice daily, and these were unchanged at discharge. Following discharge, the patient continued with an extended course of ECT, three times weekly. She remained on lorazapam/trazodone/trofinetide during this time. Taper of ECT to 2x/week was trialed on two occasions in the first several months, with worsening of symptoms noted at each trial; thus, decision made to extend the course of intensive ECT 3x/week.

Treatment has resulted in significant improvements in self-injurious behaviors, agitation, and overall engagement. The patient has been noted to be more aware of surroundings, communicating needs by pointing, and responsive to name. She is no longer noted to have extended periods of anger or crying. The patient has now completed a total of 30 ECT treatments with ongoing intensive ECT three times weekly.

## 2. Discussion

Catatonia has been documented in association with a wide range of disorders, emerging in psychiatric disorders (including psychotic disorders, mood disorders, and substance-induced disorders), medical and neurological disorders (including brain structural damage, seizure disorders, autoimmune, and endocrine disorders), developmental disorders (including autism spectrum disorder, childhood disintegrative disorder, Down syndrome, Prader–Willi syndrome), and other disorders, including anti- N-methyl-D-aspartic acid receptor encephalitis, Klein–Levin syndrome, and pervasive refusal syndrome [2].

Catatonia presents with two main subtypes. Retarded catatonia is characterized by stupor, immobility, mutism, rigidity, and withdrawal, as well as negativism, posturing, and echolalia/echopraxia. Excited catatonia is primarily characterized by psychomotor agitation and occasionally self-injurious behaviors. Though the pathophysiology of catatonia remains poorly understood, treatment with benzodiazepines is effective in many cases, with electroconvulsive therapy indicated in cases of poor response [3].

Rett syndrome has been associated with behavioral abnormalities, in particular autism spectrum disorder. It has also been associated with anxiety and mood disturbances, screaming episodes, crying, and repetitive self-injury [4]. Self-injury is a feature of both catatonia and Rett syndrome. Notably, self-injury in Rett syndrome is more prevalent in individuals with lower severity of disability [5]. Self-injury is considered a stereotypy in the context of catatonia, and the emergence of self-injury has previously been suggested as a sign of emerging catatonia in autism [6].

In this case, agitation and self-injury served as a sign that led to identification and treatment of catatonia by ECT. In conclusion, catatonia should be considered in individuals with Rett syndrome who present with agitation and self-injury to aid in overall symptom improvement and outcome.

## Data Availability

The data that support the findings of this study are available upon request from the corresponding author. The data are not publicly available due to privacy or ethical restrictions.
